# Skeletal maturation in individuals with Down's syndrome: Comparison between PGS curve, cervical vertebrae and bones of the hand and wrist

**DOI:** 10.1590/2176-9451.19.4.058-065.oar

**Published:** 2014

**Authors:** Glauber Carinhena, Danilo Furquim Siqueira, Eduardo Kazuo Sannomiya

**Affiliations:** 1 PhD in Orthodontics, School of Dentistry — University of São Paulo/Bauru.; 2 MSc in Orthodontics, Methodist University of São Paulo (UMESP).

**Keywords:** Down's syndrome, Cervical vertebrae, Age determination by skeleton, Sesamoid bones

## Abstract

**Introduction:**

This study was conducted with the aim of adapting the methods developed by Martins and Sakima to assess skeletal maturation by cervical vertebrae in the pubertal growth spurt (PGS) curve. It also aimed to test the reliability and agreement between those methods and the method of hand and wrist radiograph when compared two by two and all together.

**Methods:**

The sample comprised 72 radiographs, with 36 lateral radiographs of the head and 36 hand-wrist radiographs of 36 subjects with Down's syndrome (DS), 13 female and 23 male, aged between 8 years and 6 months and 18 years and 7 months, with an average age of 13 years and 10 months.

**Results and Conclusions:**

Results revealed that adapting the methods developed by Martins and Sakima to assess skeletal maturation by cervical vertebrae in the curve of PGS is practical and useful in determining the stage of growth and development of individuals. The stages of maturation evaluated by cervical vertebrae and ossification centers observed in radiographs of the hand and wrist were considered reliable, with excellent level of agreement between the methods by Hassel and Farman as well as Baccetti, Franchi and McNamara Jr and Martins and Sakima. Additionally, results revealed an agreement that ranged between reasonable to good for the three methods used to assess the skeletal maturation, showing statistical significance.

## Abstract (pt)

**Introdução:**

esse estudo foi realizado com o propósito de adaptar os métodos para avaliação da maturação óssea por meio das vértebras cervicais na curva do Surto de Crescimento Puberal (SCP)de Martins e Sakima, assim como testar a confiabilidade e a concordância existente entre eles e o método para a radiografia de mão e punho, quando comparados 2 a 2, e entre todos, conjuntamente.

**Métodos:**

a amostra constou de 72 radiografias, sendo 36 telerradiografias em norma lateral da cabeça e 36 radiografias de mão e punho, de 36 indivíduos com Síndrome de Down (SD), sendo 13 do sexo feminino e 23 do masculino, na faixa etária entre oito anos e seis meses até 18 anos e sete meses, com média de 13 anos e dez meses.

**Conclusão:**

de acordo com os resultados obtidos, concluímos que as adaptações dos métodos para a avaliação da maturação óssea por meio das vértebras cervicais na curva de SPC de Martins e Sakima resultaram em uma ferramenta prática e útil na determinação do estágio de crescimento e desenvolvimento dos indivíduos. Os estágios de maturação avaliados por meio das vértebras cervicais e os centros de ossificação observados nas radiografias de mão e punho foram considerados confiáveis, com excelente grau de concordância entre os métodos de diversos outros autores, e uma concordância de razoável a boa entre os três métodos para avaliação da maturação óssea quando comparados conjuntamente, sendo estatisticamente significativa.

## INTRODUCTION

The literature does not reach a consensus regarding the use of chronological age to estimate the start and end of facial growth. In other words, it is not considered a reliable parameter to assess the stage of pubertal growth of an individual. Skeletal maturation is influenced by constitutional-genetic, hormonal, nutritional, socioeconomic, climatic and seasonal as well as biochemical-pharmacological factors, which may delay or speed up due to several diseases. The Down's syndrome is among the most frequent causes of skeletal age retardation.^22^

Over the past years, the interest in studying individuals with chromosome 21 trisomy or Down's syndrome (DS) has increased. The pattern of skeletal maturation in individuals with DS has been widely investigated because the reports on the bone age of these individuals are controversial.^4,18^ The literature reports methods that are employed to determine the biological age of individuals without Down's syndrome; however, it is not known for certain the validity of these methods in a syndromic population.

The methods considered as reliable references to identify the stages of maturation during the pubertal growth spurt (PGS) are hand and wrist radiography,^6,8,13,23^ lateral cephalometric radiograph,^3,17,21^ or both.^2,19^

In 1949, Greulich and Pyle^13^ observed variations in the bone structures revealed by 60 radiographs of the hand and wrist, from birth to adulthood. This study originated an atlas that included the average data of alterations and provided the parameters of normality that serve as the basis for research and diagnostics of ossification centers. In addition, Fishman^8^ proposed a method for radiographic evaluation of the Skeletal Maturation Index (SMI) of which 11 indicators are evinced during adolescence. The sequence of the four stages of maturation, which proved stable, progressed by the increase in width of the selected epiphyseal, ossification of the adductor sesamoid, capping of the epiphysis over the shafts and finally their merging. Martins and Sakima^23^ advocated the graph of the PGS curve with the sequence of mineralization phase of ossification centers of the hand and wrist, determining whether the rate of growth was in ascending or descending phase.

Hassel and Farman^17^ assessed the stage of skeletal maturation of the cervical vertebrae and proposed a variation of the method advocated by Lamparski,^21^ correlating lateral cephalometric radiographs with radiographs of the hand and wrist. As a result, they established six stages of maturation and concluded that it is possible to determine reliable positions in relation to the degree of skeletal maturation in cervical vertebrae and the potential for future growth of individuals. Reproducibility of this method was proved by Santos et al,^29^ thus corroborating the results by Hassel and Farman.^17^ Baccetti, Franchi and McNamara Jr,^3^ who described a new version for the review of the Stages of Maturity in these bones in order to detect the moment when an individual is at the peak of mandibular growth. Their method was based on the changes in size and shape of the vertebral body, and establishes five stages of maturation instead of six, as in the method of Hassel and Farman.^17^

In the literature, there are studies reporting the level of agreement between the ossification centers of the hand and wrist and the cervical vertebrae maturation (CVM). These studies obtained statistically significant results proving the reliability of the tested methods.^2,19,30^

Although the indexes for each stage of skeletal maturation are estimated, the issue related to the type of classification exists. In other words, the analysis by Martins and Sakima^23^ enables one to determine the individual's exact location in the PGS curve, what does not occur in the subjective methods of CVM by Hassel and Farman^17^ as well as Baccetti, Franchi and McNamara Jr,^3^ both of which allow the pubertal growth stage to be estimated. Therefore, this study proposes an adaptation of the methods developed by Martins and Sakima^23^ used to assess cervical vertebrae maturation (CVM) in the PGS curve, as well as to determine reliability and agreement among the methods when compared two by two and all together.

## MATERIAL AND METHODS

The sample comprised 72 radiographs, 36 lateral cephalometric radiographs and 36 radiographs of hand and wrist from 36 individuals with Down's syndrome aged between 7 years and 8 months and 18 years and 9 months. Methods were based on the agreement analysis of three distinct methods used to assess skeletal maturation: Martins and Sakima,^23^ for hand and wrist radiographs; Hassel and Farman^17^ as well as Baccetti, Franchi and McNamara Jr^3^ for lateral cephalometric radiographs by means of cervical vertebrae observation.

For each one of the CVM assessment methods^3,17^ the subjects were classified according to their maturation stage or index. Nevertheless, all methods classified each phase of maturation differently, that is, the first comprises six stages of maturation, whereas the second comprises five stages. Therefore, adjustments were made ​​in order to visualize each method in the PGS curve, as well as in the method developed by Martins and Sakima,^23^ allowing statistical analysis to be carried out with the same type of classification.

### Adaptation process to visualize the CVM by Hassel and Farman's method^17^ in the PGS curve

**Figure 2:**
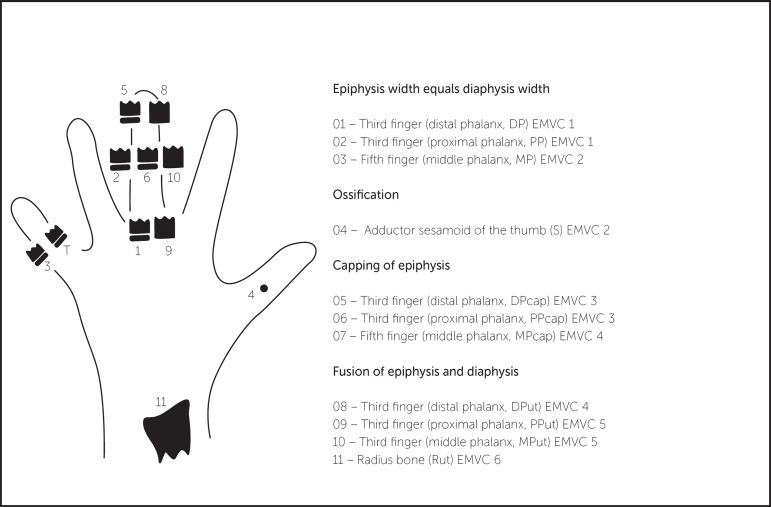
Fishman's^8^ indicators of skeletal maturation.

The stages of maturation have their own characteristics, so the morphological changes indicate different expectations of growth and development for the individual characterized by narrowing of the intervertebral space and changes in the contour of the vertebrae. Hassel and Farman^17^ separated Fishman's^8^ 11 skeletal maturation indexes (SMI) and correlated them with the shape of the contour of the cervical vertebrae (C2, C3 and C4), thus creating six distinct stages (Fig 2).

**Figure 3:**
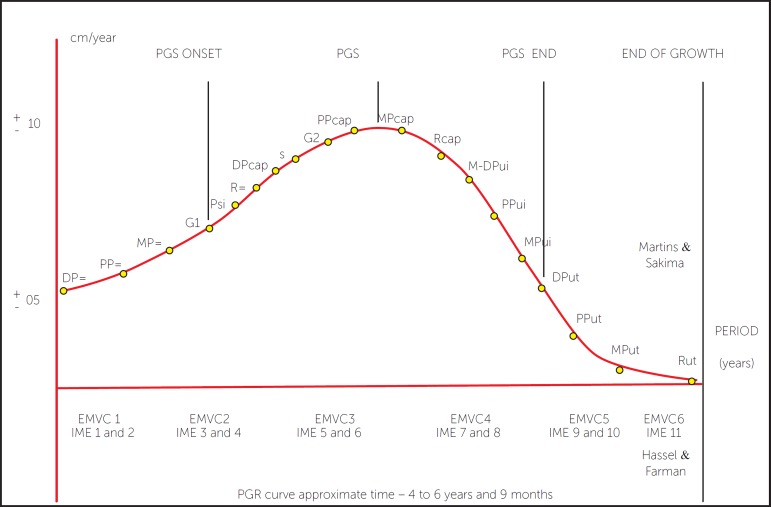
Schematic location of Hassel and Farman's^17^ stages in the PGS curve.

The adaptation of Hassel and Farman's^17^ stages was possible due to the correspondence between CVM 1 and Fishman's^8^ SMI 1 and 2, located at the beginning of Martins and Sakima's^23^ curve. Examinations performed to assess the cervical vertebrae revealed that Hassel and Farman's^17^ method comprised the highest number of stages: six stages against five for Baccetti, Franchi and McNamara Jr's.^3^ For this reason, it was necessary to divide the six stages in the PGS curve developed by Martins and Sakima^23^ (Fig 3).

### Adaptation process to visualize the CVM by Baccetti, Franchi and McNamara Jr's method^3^

**Figure 4:**
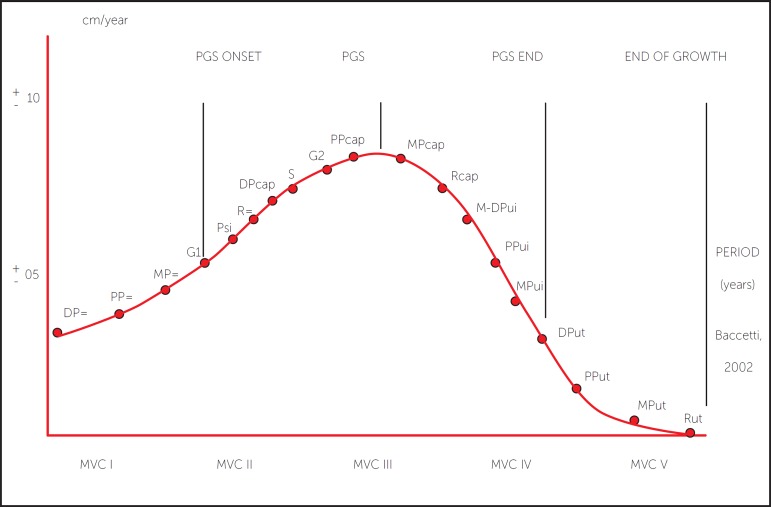
Transposing the stages by Baccetti, Franchi and McNamara Jr^3^ into Martins and Sakima's PGS curve.

Baccetti, Franchi and MacNamara^3^ proposed a new visual method which consisted on assessing the morphological characteristics of three cervical vertebrae (C2, C3 and C4) and included five stages (CVM I to V). Similarly to the method by Hassel and Farman,^17^ the five stages of Baccetti, Franchi and McNamara Jr^3^ had to be adapted in the PGS curve (Fig 4).

This adaptation was performed according to reports provided by the authors, especially with regards to the mandibular growth peak occurring between CVM II and III and which is not achieved without CVM I and II. CVM V is recorded at least two years after the growth peak. For example, the peak of mandibular growth occurs within one year after the CVM II stage. Thus, this phase ranges from G1 to the peak of PGS, and according to Martins and Sakima,^23^ the G1 phase begins 1 year before reaching the peak of PGS. Figure 4 depicts where each CVM stage is located in the pubertal growth curve.

**Figure 5:**
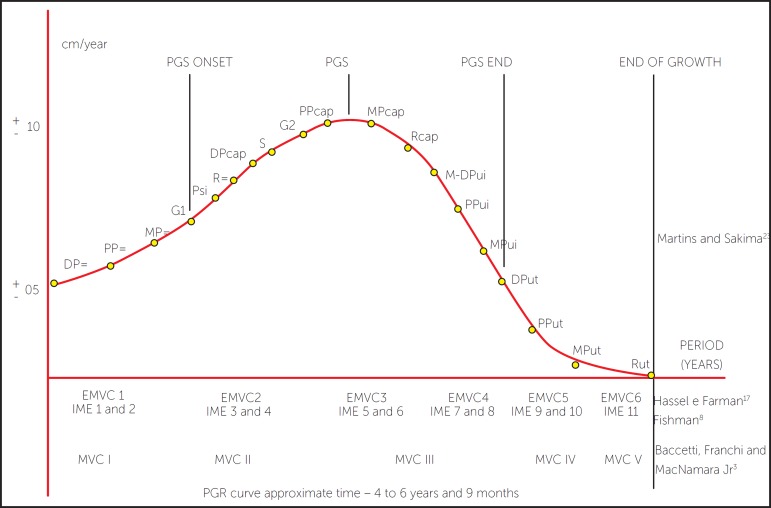
Transposing the methods in the PGS curve. Stage A - When the individual is at DP=, PP= or MP=. Stage B - When the individual is at G1, Psi, R=, DPcap, S or G2. Stage C - When the individual is at PPcap, MPcap, Rcap, M-DPui, MPui or DPut. Stage D - When the individual is at PPut or MPut. Stage E - When the individual is at Rut.

The methods used in this study allowed us to superimpose the visualization techniques of cervical vertebrae maturation over the PGS curve. New scores were assigned after dividing growth curve into five stages of ossification: A, B, C, D and E. These stages correspond to a group of ossification phenomena present in the PGS curve (Fig 5).

Two weeks after assessing lateral cephalometric radiographs (T_1_) by the methods developed by Hassel and Farman^17^ as well as Baccetti, Franchi and McNamara Jr,^3^ and after assessing the hand and wrist radiographs by the method developed by Martins and Sakima,^23^ the tests were repeated (T_2_).

Kappa agreement index was used to assess the agreement between methods, a nonparametric test. Significance level was set at 5%.

## RESULTS

Tables 1 to 3 show the results of the agreement analyses. These analyses were carried out between measurements taken at two different times (T_1_ and T_2_) with a view to assessing skeletal maturation in relation to Hassel and Farman's,^17^ Baccetti, Franchi and McNamara Jr's^3^ as well as Martins and Sakima's^23^ methods, respectively. Tables 1 to 3 also show that the three methods studied herein present a statistically significant correlation (P < 0.05) between T_1_ and T_2_, thus indicating excellent level of agreement between measurements (Kappa > 0.75). Therefore, satisfactory calibration was obtained for the classification criteria applied to the measures of each method.

**Table 1:** Error of the method assessed by means of agreement analysis for evaluation of skeletal maturation by Hassel and Farman's method.^17^

T_2_					
T_1_	A	B	C	D	Total
Total	02	10	11	13	36

Kappa agreement index = 0.76 (P < 0.0001).

**Table 3:** Error of the method assessed by means of agreement analysis for evaluation of skeletal maturation by Martins and Sakima's method.^23^

T_2_														
T_1_	DP	PP	MP	Psi	R	S	MPcap	M-DPui	MPui	DPut	PPut	MPut	Rut	Total
Total	2	1	2	1	2	3	2	1	2	3	2	12	3	36

Kappa agreement index = 0.80 (P < 0.0001).

The blue diagonal line highlighted in Tables 1 to 3 refers to cases in which both measurements agree.

Tables 4 to 6 show agreement of final results among the three methods of assessing skeletal maturation compared two by two. The data obtained show an excellent level of statistically significant (P < 0.05) agreement (Kappa > 0.75) between the methods by Hassel and Farman^17^ and Baccetti, Franchi and McNamara Jr;^3^ Hassel and Farman^17^ and Martins and Sakima;^23^ and Baccetti, Franchi and McNamara Jr^3^ and Martins and Sakima.^23^ Thus, the methods evaluated two by two are similar in terms of classification of skeletal maturation. The blue diagonal line highlighted in Tables 4 to 6 refers to cases in which both methods agree.

**Table 4:** Agreement analysis between the methods by Hassel and Farman,^17^ and Baccetti, Franchi and McNamara Jr^3^ for evaluation of skeletal maturation.

	Baccetti, Franchi and Macnamara Jr^3^					
Hassel and Farman^17^	A	B	C	D	E	Total
Total	02	10	11	13	0	36

Kappa agreement index = 0.80 (P < 0.0001).

**Table 6:** Agreement analysis between the methods by Baccetti, Franchi and McNamara Jr,^3^ and Martins and Sakima^23^ for evaluation of skeletal maturation.

Martins and Sakima^23^						
Baccetti, Franchi and MacNamara^3^	A	B	C	D	E	Total
Total	05	08	07	13	03	36

Kappa agreement index = 0.81 (P < 0.0001).

Table 7 shows that there is a statistically significant correlation that ranges from reasonable to good (0.40 < Kappa < 0.75) when the three methods used to assess skeletal maturation are compared all together.

**Table 7:** Agreement analysis between the methods by Hassel and Farman,^17^ Baccetti, Franchi and McNamara Jr,^3^ and Martins and Sakima^23^ compared all together for evaluation of skeletal maturation.

Response						
Individuals	A	B	C	D	E	Total
Total	9	28	30	38	3	108

Kappa agreement index = 0.63 (P < 0.0001).

## DISCUSSION

The process of skeletal maturation is directly related to height, speed and specific amounts of craniofacial growth; however, no pattern can be established on the basis of simple chronology, only. Every individual undergoes a particular sequence of events and, for this reason, generalizing the descriptions of maturation stages associating them with the skeletal growth curve determined for the population as a whole can lead to error. Therefore, the concept of "normal skeletal age" should be questioned and the individuality of diagnosis should be valued.^9^

Several parameters are employed to predict the stage in which an individual is on the growth curve, namely: Chronological, dental and circumpubertal ages, which not only consider the emergence of secondary sexual and skeletal characteristics, but also the height-weight ratio. Since an individual's chronological age is not reliable to determine the beginning and end of facial growth, the skeletal age should be determined to define the individual's stage of biological growth, given that it proves to be the most reliable parameter for biological evaluation.^6,20,27,29^

Skeletal maturation is influenced by constitutional-genetic, hormonal, nutritional, socioeconomic, climatic, seasonal, as well as biochemical-pharmacological factors, which may delay or speed up due to the presence of several diseases. Down's syndrome is among the most frequent causes of skeletal age retardation.

The pattern of skeletal maturation in individuals with DS has been widely investigated because the reports on the skeletal age of these individuals are controversial.^4,15,18^ According to Marcondes,^22^ the concept of bone age does not apply to newborns (non-carriers of chromosome 21 trisomy), given that the first carpal core is only observed after the third month. This finding confronts the studies by Hall,^16^ which claims to be possible to determine bone age at this stage of life by means of the ossification centers of individuals with DS.

According to the literature, the stage of maturation is influenced by factors such as sex, race, ethnic groups, among others. Bone development and growth were reported by Prates, Peters and Lopes^24^ as well as Guzzi and Carvalho^14^ who assessed skeletal maturation using the method by Greulich and Pyle.^13^ The authors observed that in non-syndromic females individuals aged between 13 and 14 years, as well as between 9 and 16 years old, respectively, an index of accelerated maturation was found.

As for male patients, Guzzi and Carvalho^14^ found skeletal maturation retardation in non-syndromic individuals, which was also observed by Aguiar^1^ as well as Sannomiya and Calles.^26^ who compared non-syndromic patients with individuals with Down's syndrome aged between 5 and 19 years old. Furthermore, it should be emphasized that the method proposed by Eklöf and Ringertz^7^ was not considered reliable to assess skeletal maturation in this population.

An individual's chronological moment may be used to determine one's bone age, provided that certain parameters be respected.^12^ Franchi, Baccetti and McNamara Jr^10^ observed a significant decrease between stages 4 and 5^3^ after the end of pubertal growth. They further highlighted that this is a reliable method in the assessment of skeletal maturation. Canali, Brücker and Lima^5^ as well as Generoso et al^12^ reported potential direct relationship between chronological age and CVM; however, skeletal maturation in female patients occurs earlier (about 1 year).

The preference and choice regarding the different methods are based on the experience and technical training of each professional. In addition, the reliability of the method consists of its ability to be compared, which is verified by intra-observer testing; as well as its reproducibility, observed by inter-observer assessment. In this study, the method proposed by Martins and Sakima^23^ was used for hand and wrist radiographs, based on centers of ossification, whereas the methods of Hassel and Farman^17^ as well as Baccetti, Franchi and McNamara Jr^3^ were used for lateral cephalometric radiographs.

Radiographs were assessed and skeletal maturity stages were determined by a single observer, properly calibrated. Initially, the error of the method was observed at two different times (T_1_ and T_2_), based on the analysis of new scores attributed to the hand and wrist radiographs and the lateral cephalometric radiographs, as shown in Tables 1 to 3. Table 1 shows agreement in bone assessment using the method by Hassel and Farman,^17^ with Kappa index statistically significant (p < 0.05), thus indicating excellent level of agreement between measurements (Kappa > 0.75).

Excellent level of agreement was also observed for the methods by Martins and Sakima^23^ as well as Baccetti, Franchi and McNamara Jr^3^ of which values ​​are presented in Tables 2 and 3, respectively. Therefore, satisfactory calibration was obtained for the classification criteria applied to the measures of each method. Intra-observer assessment revealed that the scores attributed to the methods by Martins and Sakima^23^ as well as Hassel and Farman^17^ agreed in 30 out of 36 subjects (83.3 %); whereas for Baccetti, Franch and McNamara Jr^3^ there was an agreement of 32 out of 36 subjects (88.8 %). This percentage difference in favor of the latter may be due to greater assimilation of the operator, perhaps because it is a method of classification with fewer steps and, therefore, less subjective.

**Table 2:** Error of the method assessed by means of agreement analysis for evaluation of skeletal maturation by Baccetti, Franchi and McNamara Jr's method.^3^

T_2_					
T_1_	A	B	C	D	Total
Total	02	10	12	12	36

Kappa agreement index = 0.84 (P < 0.0001).

The results obtained from the lateral cephalometric radiographs analyzed by the method proposed by Hassel and Farman,^17^ as reported by Santos and Almeida,^30^ Canali, Brücker and Lima^5^ as well as Santos et al,^29^ showed a positive and significant correlation, thus indicating that the scores attributed to each one of them were similar.

Table 2 shows a positive and significant correlation for the comparison between T_1_ and T_2_, which agrees with the two observers used in the study by Baccetti, Franchi and McNamara Jr.^3^ The error of the method analysis proposed by Martins and Sakima^23^ was performed by Iguma, Tavano and Carvalho^20^ who found a high correlation when assessing the PGS. Their study also found excellent agreement as revealed by the Kappa index obtained for the sample studied.

Should, in fact, there be an association between the aforementioned methods and the hand and wrist as well as the cervical vertebrae, this means that it would be possible to choose one of them to assess patient's skeletal maturation for routine orthodontic records. To elucidate a possible correlation between the methods proposed in this study, an agreement analysis of the final results was conducted by comparing the methods two by two.

The data obtained showed that the level of agreement between the methods by Hassel and Farman^17^ and Baccetti, Franchi and McNamara Jr^3^ (Table 4); Hassel and Farman^17^ and Martins and Sakima^23^ (Table 5); as well as Baccetti, Franchi and McNamara Jr^3^ and Martins and Sakima^23^ (Table 6) were statistically significant with excellent level of agreement between them. Table 4 reveals that scores were concordant in 33 out of 36 subjects (91.6 %). Tables 5 and 6 reveal that 23 out of 36 subjects (63.8 %) and 25 out of 36 (69.4 %) were concordant, respectively. Data presented in Table 4 suggest that the lower the degree of subjectivity among the methods used, the higher the index of agreement, since both methods use inspection parameters based on the size and shape of the vertebrae.

**Table 5:** Agreement analysis between the methods by Hassel and Farman,^17^ and Martins and Sakima^23^ for evaluation of skeletal maturation

Martins and Sakima^23^						
Hassel and Farman^17^	A	B	C	D	E	Total
Total	05	08	07	13	03	36

Kappa agreement index = 0.77 (p < 0.0001).

The literature includes studies that report the use of agreement analysis between two different methods. Garcia^11^ as well as Santos and Almeida^30^ employed Fishman's^8^ methods for hand and wrist, whereas Hassel and Farman^17^ used it for cervical vertebrae and noted statistically significant correlation between them. San Román et al^28^ confirm the previous results; however, the authors used the Grave and Brown method for hand and wrist.

It is observed that there is a difference regarding the choice of which method to use to assess hand and wrist as well as cervical vertebrae. However, regardless of the method studied, the results were similar, thus suggesting a correlation between maturation of vertebral bones and hand and wrist.

Table 7 shows a statistically significant correlation that ranges from reasonable to good (0.40 < Kappa < 0.75) among the three methods proposed to assess skeletal maturation when they were compared all together. Out of the 36 subjects assessed, 22 (61.1 %) achieved the same score for all three methods of bone maturation, whereas 14 (38.9 %) were not in agreement and 12 differed in only one stage with a difference of 1 score (the subject was "A" for a particular assessment method and "B" for another). One individual got different scores for all methods, and despite agreeing with the methods by Baccetti, Franchi and McNamara Jr^3^ as well as Hassel and Farman,^17^ one subject obtained a difference of two scores in relation to the method by Martins and Sakima.^23^

According to Hassel and Farman,^17^ the 12 non-coincident results that varied in only one contiguous score have no clinical relevance to invalidate the method; and, for this reason, these results should be considered acceptable. Many dubious cases may not allow a stage to be determined with precision, especially if one considers that the radiograph may have been obtained in a phase of transition from one stage to another subsequent. Thus, the examiner can classify the individual both in the beginning of a certain stage or in the end of another. We also emphasize that if these 12 individuals were considered acceptable, we would obtain an excellent Kappa agreement index.

One of the most important factors in assessing the stage of maturation by means of hand and wrist as well as lateral cephalometric radiographs was the presence of 19 ossification centers used to place the individual in the PGS curve by means of the method advocated by Martin and Sakima,^23^ when compared to the methods by Baccetti, Franchi and McNamara Jr^3^ (five stages) as well as Hassel and Farman^17^ (six stages). For this reason, the method by Martins and Sakima^23^ proves more subjective, given the difference in scores observed in 10 out of 12 non-concordant individuals for the three methods of the sample.

## CONCLUSIONS

Based on the results of this study it is reasonable to conclude that:

» Adapting the methods developed by Martins and Sakima^23^ to assess skeletal maturation by cervical vertebrae in the curve of PGS is a practical and useful tool in determining the stage of growth and development of individuals.

» Stages of maturation assessed by cervical vertebrae and ossification centers observed in radiographs of the hand and wrist were considered reliable.

» The data obtained revealed an excellent level of agreement between the methods by Hassel and Farman^17^ and Baccetti, Franchi and McNamara Jr,^3^ Hassel and Farman^17^ and Martins and Sakima,^23^ as well as Baccetti, Franchi and McNamara Jr^3^ and Martins and Sakima,^23^ all of which were statistically significant;

» A statistically significant correlation that ranged from reasonable to good was obtained among the three methods used to assess skeletal maturation when they were compared all together.

## Figures and Tables

**Figure 1:**
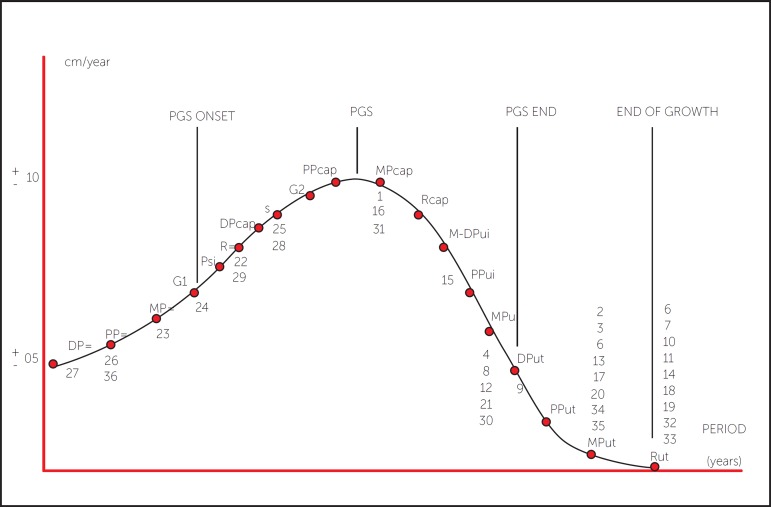
Graphical representation of individuals distributed in the PGS curve.
